# Unplanned pregnancies and contraceptive use among HIV- positive women in care

**DOI:** 10.1371/journal.pone.0197216

**Published:** 2018-05-17

**Authors:** Madeline Y. Sutton, Wen Zhou, Emma L. Frazier

**Affiliations:** 1 Division of HIV/AIDS Prevention, National Centers for HIV, Viral Hepatitis, STD and TB Prevention, Centers for Disease Control and Prevention, Atlanta, GA, United States of America; 2 Department of Obstetrics and Gynecology, Morehouse School of Medicine, Atlanta, GA, United States of America; 3 ICF, Atlanta, GA, United States of America; Massachusetts General Hospital, UNITED STATES

## Abstract

Among 230,360 women with diagnosed HIV in the United States (U.S.), ~ 8,500 give birth annually, and unplanned pregnancies (as with HIV-negative women) are prevalent. However, unplanned pregnancies and contraceptive use among HIV-positive women have been understudied. To examine unplanned pregnancies and contraceptive use among HIV-positive women, we used 2013–2014 data from the Medical Monitoring Project (MMP), an HIV surveillance system that produces national estimates for HIV-positive adults in care in the U.S. (Pregnancy outcome dates were from years 1986–2015 for this cohort of women who were interviewed during 2013–2014; median year of reported pregnancy outcome was year 2003). Women in HIV care and diagnosed with HIV before age 45 (reproductive age) were included. We calculated adjusted prevalence ratios (aPR) of unplanned pregnancies with 95% confidence intervals (CI). For women who were aged 18–44 years at time of interview, we computed weighted prevalences of contraceptive use (previous 12 months) by method, including permanent (i.e., sterilization), short-acting (i.e., pills, depo-progesterone acetate (DMPA)), long-acting reversible contraceptives (LARC) (i.e., implants), and barriers (i.e., condoms). Six hundred seventy-one women met criteria for the unplanned pregnancy analysis; median age at HIV diagnosis = 24.6 years, and 78.1% (CI:74.5–81.7) reported ≥ 1 unplanned pregnancy. Women reporting unplanned pregnancies were more likely to be non-Hispanic white (aPR = 1.20; CI 1.05–1.38) or non-Hispanic black (aPR = 1.14; CI 1.01–1.28) than Hispanic, to be above the poverty level (aPR = 1.09; CI 1.01–1.18), and to have not received care from an OB/GYN in the year before interview (aPR = 1.13; CI 1.04–1.22). Among 1,142 total pregnancies, 795 (69.6%) were live births; 70 (7.8%) were born HIV-positive; 42 (60%) of those born HIV-positive were unplanned pregnancies. For the contraceptives analysis (n = 957 women who were aged 18–44 at time of interview), 90.5% reported using at least one contraceptive, including 59.7% reporting barrier methods, 29.9% reporting permanent sterilization, and 22.8% reporting short-term methods in the previous year. LARC was used by only 5.3% of women. Women who reported use of LARC or DMPA were more likely to be aged 18–29 years (aPR = 3.08; CI 1.61–5.89) or 30–39 years (aPR = 2.86; CI 1.76–4.63) compared with women aged 40–44 years. Unplanned pregnancies were prevalent and LARC use was low; prevention efforts should strengthen pregnancy planning and contraceptive awareness for HIV-positive women during clinical visits.

## Introduction

Women comprised 27% of adults living with diagnosed HIV infection in the United States (U.S.) as of 2014 [1]. Of women diagnosed with HIV in 2015, 64.7% were of reproductive age (aged 18–44 years), and heterosexual activity remained the primary mode of HIV transmission [2]. Also, nearly 8,500 HIV-positive women give birth annually [1, 2]. Although overall rates of unplanned pregnancies are declining, they account for at least 45% of pregnancies among women in the U.S.; this suggests room for improvement in contraception awareness, access, utilization, and pregnancy planning, [3, 4] and also some progress toward the Healthy People 2020 goal of 56% of all pregnancies being planned or intended [5]. Despite progress made in recent years with increased contraceptive options, including longer-acting, reversible contraceptives (LARC) with strong safety and efficacy data, [6] unplanned pregnancies remain a major public health challenge domestically and abroad [3,7,8]. For HIV-positive women who may not be in treatment, unplanned pregnancies are of particular concern, because of potential missed opportunities for early access to antiretroviral treatment to decrease maternal morbidity and maternal-to-child HIV transmission [9, 10]. Yet, the frequency and depth of discussions between HIV-positive women and their health care providers regarding pregnancy intentions and contraception are suboptimal [11].

Despite data showing contraceptive efficacy for HIV-positive women,[12] access to and utilization of the most effective contraceptive methods remain low [13]. The Women’s Interagency HIV Study (WIHS) reported that 37 of 213 (17.4%) HIV-positive women were using highly effective contraception (i.e., pills, injections, implants and intrauterine devices [IUDs]), including 6 of 213 (2.8%) using LARC [13]. Compared with HIV-negative women, LARC use was lower among HIV-positive women and was not associated with consistent condom use; reasons for this were unclear [13].

Because unplanned pregnancies and contraceptive use remain understudied for HIV-positive women, we examined unplanned and planned pregnancies among HIV-positive women and past-year contraceptive use among HIV-positive women, to inform and strengthen prevention strategies and clinical care.

## Materials and methods

### Data

For this combined cross-sectional study, we analyzed matched interview and medical record abstraction data from the 2013 and 2014 data collection cycles of the Medical Monitoring Project (MMP), an HIV surveillance system which produces annual nationally representative estimates of characteristics of adults living with HIV and receiving medical care in the U.S. Briefly, the MMP cycles used a three-stage, probability-proportional-to-size sampling method. First, U.S. states and one territory were sampled, then facilities in those areas providing outpatient HIV care, and finally, eligible HIV-infected patients. MMP methods, including non-response bias analysis and weighting techniques, have been described in detail elsewhere [14, 15].

### Participants and measures

Eligible persons were HIV-infected, age 18 years or older, and had received medical care in participating facilities between January and April in the cycle year for which they were sampled. Data were collected from 23 health jurisdictions [California, Chicago (IL), Delaware, Florida, Georgia, Houston (TX), Illinois, Indiana, Los Angeles County (CA), Michigan, Mississippi, New Jersey, New York state, New York City, North Carolina, Oregon, Pennsylvania, Philadelphia (PA), Puerto Rico, San Francisco (CA), Texas, Virginia, and Washington state] were funded and collected data from June 2013 through May 2015 (2013–2014 data cycles). During these data cycles, the average facility and response rates, adjusted for eligibility were 86% (range = 85–86%) and 56% (range = 55–56%), respectively.

We combined MMP 2013–2014 data cycles (n = 10,184); 27% (n = 2,766) were women. We limited our unplanned pregnancy analysis sample to women diagnosed as HIV-positive at age ≤ 45 years (reproductive years) (n = 2,228), and women who reported having had at least one planned or unplanned pregnancy after HIV diagnosis. Pregnancy outcome dates were from years 1986–2015 for this cohort of women who were interviewed during 2013–2014; median year of reported pregnancy outcome was year 2003. We excluded 15 women because they did not respond about whether they had any pregnancies (n = 13) or if they had any unplanned pregnancies (n = 2), resulting in a final sample size for this analysis of 671 women. All women were asked about the number of pregnancies after learning of HIV diagnosis (“How many times have you been pregnant after you learned you had HIV?”). Women who reported one or more pregnancies after HIV diagnosis were asked if they were trying to get pregnant for each pregnancy for the first through fifth pregnancies (“For each pregnancy after you learned you had HIV, were you trying to get pregnant?”). Women who responded “no” to this question were classified as having an unplanned pregnancy [16, 17]; otherwise, women were considered as having had only planned pregnancies. We defined any unplanned pregnancy as having ≥ one unplanned pregnancy among all pregnancies occurring after learning of an HIV diagnosis. Women also reported the outcome (currently pregnant, live birth, stillbirth, miscarriage, abortion) and date each outcome occurred for each of the first through fifth pregnancies. If the outcome of the pregnancy was a live birth, women reported whether vertical transmission occurred (“Was the child diagnosed with HIV?”). Because HIV management of HIV-positive pregnant women to reduce mother-to-child transmission of HIV evolved quickly after 1994, [18] and because diagnoses of perinatally acquired HIV infection in infants decreased significantly from 2002–2013, [19] we reviewed birth outcomes by year of pregnancy (before 2004; during and after 2004).

Socio-demographic variables collected included age at HIV diagnosis, age at interview, length of time since HIV diagnosis, race/ethnicity, highest educational attainment and being foreign born. The following variables were reported by respondents based on the 12 months prior to being interviewed: homelessness; type of health insurance coverage, whether received public assistance, and whether living below poverty level. The number and percentage of participants meeting current poverty guidelines were determined using the U.S. Department of Health and Human Services poverty level [20] that corresponded to the calendar year for the combined household income.

The following behavioral variables were reported based on the 12 months prior to interview and were included as factors that may be relevant for any woman living with HIV infection: any drug use (both injection and non-injection) for non-medical purposes, any sexual activity with a male, HIV status of current or most recent partner, and sex risk with a partner, including any condomless sex with a male partner who was HIV-negative or of unknown status.

Women reported several care indicators that occurred in the previous 12 months: receipt of sexually transmitted infections (STI) prevention counseling by a health care professional, whether they were taking antiretroviral therapy (ART), and whether they had seen an obstetrician (OB) or gynecologist (GYN). Additionally, clinical care information was abstracted from participants’ medical records for the year prior to interview, and included the following: prescription of ART; number of CD4 cell count or viral load tests; sustained viral suppression (defined as all viral loads in the past 12 months as undetectable or < 200 copies/ml); and disease stage. The HIV disease stage was determined per Centers for Disease Control and Prevention (CDC) criteria (Stage 1: No AIDS and nadir CD4 ≥ 0.500 x 109 cells/L [or CD4% ≥ 29]) Stage 2: No AIDS and nadir CD4 0.200–0.499 x 109 cells/L [or CD4% 14-< 29], or Stage 3: AIDS or nadir CD4 0–0.199 x 109 cells/L [or CD4% < 14]) [21].

### Data analyses

Weighted estimates of percentages and associated 95% confidence intervals (CIs) were computed to describe the characteristics of women in our sample. We used the design-based Rao Scott chi-square test to estimate the percentages of pregnancy categories after HIV diagnosis by key characteristics [22]. Demographic characteristics were summarized using medians and interquartile ranges (IQRs); weighted prevalence estimates were computed for categorical data.

We performed logistic regression to compute unadjusted and adjusted prevalence ratios (aPR) and CIs. Multivariable logistic regression was used to calculate aPR, based on predicted marginals, and CIs for correlates of unplanned pregnancies among women with ≥ 1 pregnancy. Factors associated with having had an unplanned pregnancy at p < 0.10 and those based on any a priori information from the literature [4, 11] were used for initial inclusion in the multivariable regression models. The final full unplanned pregnancies model included all variables from the bivariate analysis that were significant at the p < 0.10 level.

We additionally examined contraceptive use in the past year among HIV-positive women aged 18–44 years at the time of MMP interview (n = 957) and compared those who were users of LARC and depo-medroxyprogesterone acetate (DMPA) (long-acting and non-daily or non-weekly administered contraceptives) to those who used other forms of contraception. (Though oral contraceptives (OCPs) are considered highly effective [perfect use failure rate < 99.9%], because they require daily compliance for optimal success, we did not group them with LARC and DMPA for this analysis). Women reported if any birth control methods were used in the past year to prevent pregnancy (“Have you used this method to prevent pregnancy in the past 12 months?”). Women could select multiple birth control methods in response to a list of sixteen methods (S1 Table) [23]. We computed weighted prevalences of contraceptive use (previous 12 months) by method, including permanent (i.e., sterilization), short-acting (i.e., pills), long-acting reversible contraceptives (LARC) (i.e., implants), and barriers (i.e., condoms). We used a hierarchical approach such that women reporting LARC or DMPA at all were placed into these categories for analysis. For this multivariable logistic regression, we also excluded women who were not able to become pregnant due to sterilization or hysterectomy. Lastly, we conducted a sensitivity analysis, which compared women with pregnancies in the past year with women with a pregnancy at any time since HIV diagnosis.

All analyses were performed using SAS 9.3 (SAS Institute, Cary, NC) and SAS-callable SUDAAN 10.0.1 (RTI International, Research Triangle Park, NC) and accounted for clustering, unequal selection probabilities, and non-response.

In accordance with guidelines for defining public health research, [24] MMP was determined by the National Center for HIV, Viral Hepatitis, STD and TB Prevention’s Office of the Associate Director of Science at the Centers for Disease Control and Prevention (CDC) to be a non-research, public health surveillance used for disease control, program, or policy purposes. MMP is not subject to human subjects regulations, including federal investigational review board (IRB) review. However, local IRB approval was obtained at participating states, territory, and facilities when required locally. Individual signed, written informed consents were obtained locally from all interviewed adult participants, aged ≥18 years. In addition, access to any information that would directly identify individual persons on whom data were collected were not available to any of the authors of this manuscript (de-identified data).

## Results

Selected characteristic data for the full sample of 2,228 women diagnosed as HIV-positive before age 45 years (no pregnancies and any pregnancies) are shown in the Supplementary Table (S2 Table). Of those, 1,557 of 2,228 (69.8%) had no pregnancies. There were 671 of 2,228 (30.2%) women with at least one pregnancy (for a total of 1,142 pregnancies after diagnosis); their median age at diagnosis was 24.6 years (IQR = 20.3–29.1). Of the 671 women, 524 (78.1%) (median age at diagnosis 24.4 years [IQR = 20.0–28.8]) reported at least one unplanned pregnancy (Table 1). (S1 Fig shows a flow chart for the analytic samples).

**Table 1:** Selected characteristics of HIV-positive women in care who had a pregnancy since HIV diagnosis, by only planned pregnancies vs. ≥ 1 unplanned pregnancies—Medical Monitoring Project—2013–2014 (N = 671).

Characteristics	Had only planned pregnancies^1^^†^		Had ≥1 unplanned pregnancies^1^^†^		Chi-square
	n	%(95% CI)	n	%(95% CI)	p-value^
**Total**	147	21.9 (18.3–25.5)	524	78.1 (74.5–81.7)	
**Age group at time of diagnosis (years) **					0.55
≤19	18	13.1 (7.7–18.5)	91	17.8 (13.9–21.7)	
20–24	41	26.6 (19.9–33.4)	151	29.7 (25.9–33.4)	
25–29	44	29.9 (22.5–37.4)	147	28.5 (24.9–32.0)	
30–34	31	21.7 (16.0–27.5)	92	16.7 (13.3–20.1)	
35–39	10	6.3 (1.9–10.8)	34	5.8 (3.6–7.9)	
40–44	3	2.3 (0.0–5.1)	9	1.7 (0.3–3.0)	
**Age group at time of interview (years)**					0.80
18–29	19	13.4 (7.7–19.1)	63	12.7 (8.9–16.6)	
30–39	43	29.5 (23.2–35.8)	147	27.8 (23.8–31.9)	** **
40–44	33	18.1 (11.8–24.4)	119	22.1 (17.7–26.4)	** **
≥45	52	38.9 (31.2–46.6)	195	37.4 (32.8–41.9)	** **
**Race/Ethnicity**					**<0.01**
Non-Hispanic black	87	56.7 (45.9–67.5)	312	60.6 (52.1–69.0)	** **
Non-Hispanic white	15	8.7 (3.3–14.0)	104	16.5 (12.1–20.9)	** **
Hispanic**	45	32.6 (22.0–43.1)	108	19.2 (10.2–28.1)	** **
Other	3	2.1 (0.0–4.6)	17	3.8 (1.5–6.0)	** **
**Highest Educational Attainment**					0.67
<High school	40	31.2 (21.9–40.6)	148	27.4 (23.0–31.9)	
High school graduate	49	31.9 (23.2–40.6)	166	32.7 (28.3–37.2)	
>High school	58	36.9 (28.8–45.0)	209	39.8 (35.8–43.9)	
**Time since HIV diagnosis (years)**					0.41
<5years	13	8.5 (3.8–13.1)	42	8.7 (5.8–11.7)	
5 years -< 10 years	34	22.4 (15.7–29.1)	95	17.3 (13.7–20.9)	
≥10 years	100	69.1 (61.7–76.5)	387	73.9 (69.6–78.2)	** **
**Foreign born**	38	26.9 (17.6–36.2)	94	18.0 (13.1–22.9)	**0.02**
**Variables based on information from the 12 months prior to MMP interview**					
**Any drug use in past year**	18	12.5 (6.8–18.3)	98	18.6 (14.0–23.3)	0.08
**Homelessness**	14	9.8 (4.1–15.5)	46	9.0 (6.6–11.5)	0.80
**Received public assistance**	64	46.0 (34.1–57.8)	263	52.4 (46.6–58.1)	0.31
**At or below poverty level**	111	81.0 (74.4–87.6)	352	69.2 (63.7–74.7)	**<0.01**
**Received care from OB/GYN**	66	47.6 (39.0–56.1)	162	31.8 (24.7–38.9)	**<0.01**
**Currently taking ART**	137	94.3 (90.5–98.2)	494	93.8 (91.7–95.9)	0.82
**Health Insurance Coverage**					0.31
Any private insurance	29	18.5 (11.3–25.7)	90	17.6 (13.6–21.6)	** **
Public insurance only	96	66.4 (56.9–75.9)	374	71.7 (65.8–77.6)	** **
RW only/Uninsured	22	15.1 (7.7–22.5)	60	10.7 (7.5–13.8)	** **
**Sexually active with a male partner**	112	74.4 (67.4–81.4)	347	65.2 (60.2–70.3)	0.05
**Sex risk/partner HIV status**					0.21
Reported condomless sex with HIV-negative or unknown status male partner	26	23.8 (15.5–32.2)	115	31.9 (26.5–37.3)	** **
Reported condomless sex with HIV-positive male partner	17	14.7 (8.0–21.4)	34	10.6 (7.2–14.0)	** **
Did not report any condomless sex or not sexually active	67	61.5 (51.7–71.2)	189	57.5 (52.1–62.9)	** **
**Received STI prevention counseling by a health professional**	96	65.5 (54.9–76.1)	306	56.9 (51.2–62.6)	0.07
**Had ≥ 2 CD4 or viral load tests** ^‡^	135	92.0 (87.4–96.6)	440	84.6 (81.5–87.6)	**0.01**
**Prescribed ART**^‡^	141	96.8 (94.1–99.5)	483	92.2 (89.7–94.7)	0.06
**Had sustained viral suppression** ^‡^*	91	65.1 (55.9–74.4)	314	58.7 (54.4–63.0)	0.24
**Used barrier birth control methods (past year)**					**0.02**
No	50	36.1 (27.6–44.6)	240	47.0 (42.0–52.0)	** **
Yes	97	63.9 (55.4–72.4)	283	53.0 (48.0–58.0)	** **
**Variables based on information reported from any time since HIV diagnosis**					
**Number of Pregnancies since HIV diagnosis**					0.06
1	95	64.3 (56.0–72.6)	289	54.8 (49.5–60.2)	** **
≥2	52	35.7 (27.4–44.0)	235	45.2 (39.8–50.5)	** **
**Data cycle year**					0.30
2013	74	54.7 (46.3–63.2)	254	49.5 (44.7–54.3)	** **
2014	73	45.3 (36.8–53.7)	270	50.5 (45.7–55.3)	** **
**Women with Pregnancies since HIV diagnosis**					0.71
Before the year 2004 only	56	37.4 (28.1–46.7)	224	42.1 (36.6–47.6)	
During and after 2004 only	54	33.9 (26.3–41.4)	183	34.3 (28.5–40.2)	
Both before 2004 and during or after 2004	9	6.5 (1.9–11.1)	35	5.9 (3.6–8.1)	
No dates provided for the pregnancies	28	22.2 (12.6–31.8)	82	17.7 (9.6–25.8)	

^1^Self-reported pregnancies that occurred during or after HIV diagnosis.

Abbreviations: n = unweighted sample size; CI = Confidence interval; ART = Antiretroviral medications; VL = Viral Load; STI = Sexually Transmitted Infection; OB/GYN = Obstetrician/Gynecologist.

Time period: In the past 12 months, unless otherwise noted. All measures are self-reported unless otherwise noted.

Bold = significant at p < 0.05 level.

^†^ Weighted row percentage.

^‡^ Based on medical record abstraction data in the past 12 months prior to interview.

^^^ Chi-square p-value based on the Rao-Scott chi-square.

* Sustained viral suppression is defined as all viral loads in the last 12 months undetectable or <200 copies/ml.

**Hispanics can be of any race/ethnicity.

Of the 1142 pregnancy outcomes, 254 of 795 (31.9%) livebirths were among women who reported only planned pregnancies, and 541 of 795 (68.1%) livebirths were among women reporting any unplanned pregnancies. Of 119 abortions, 8 of 119 (6.7%) were among women who reported only planned pregnancies, and 110 of 119 (92.4%) were among women reporting any unplanned pregnancies.

In the unplanned pregnancy bivariate analyses, women with any unplanned pregnancies were more likely to be non-Hispanic black or non-Hispanic white, and to have had ≥ two pregnancies (p< 0.01). Women with only planned pregnancies were more likely to have received care from an OB/GYN, to have two or more CD4 or viral load tests in the past year, and to report use of barrier contraceptives (p<0.05). (Table 1). In multivariable analyses, women reporting any unplanned pregnancies were more likely to be non-Hispanic white or non-Hispanic black compared with Hispanic, to be living above the poverty level, and to have not received care from an OB/GYN in the past year (Table 2).

**Table 2:** Associations between selected characteristics and having had ≥ 1 unplanned pregnancy among HIV-positive women in care—Medical Monitoring Project, 2013–2014 (N = 671).

Characteristics	Unadjusted/Bivariate analyses	Adjusted/Multivariate analyses	
	PR (95% CI)	APR (95% CI)	p-value
**Race/Ethnicity**	** **	** **	**0.01**
Non-Hispanic black	1.17 (1.03–1.33)	1.14 (1.01–1.28)	** **
Non-Hispanic white/Other^1^	1.29 (1.13–1.47)	1.20 (1.05–1.38)	** **
Hispanic	Reference	Reference	
**Foreign born**	** **	** **	0.40
No	1.14 (1.00–1.29)	1.05 (0.93–1.18)	
Yes	Reference	Reference	
**Any drug use in past year**	** **	** **	0.26
No	Reference	Reference	** **
Yes	1.10 (1.00–1.20)	1.06 (0.96–1.18)	** **
Poverty level**	** **	** **	**0.04**
Above poverty level	1.13 (1.06–1.21)	1.09 (1.01–1.18)	** **
At or below poverty level	Reference	Reference	** **
**Received care from OB/GYN in past year **			**0.003**
No	1.17 (1.08–1.26)	1.13 (1.04–1.22)	
Yes	Reference	Reference	
**Sexually active with a male partner in past year **			0.51
No	1.09 (1.01–1.19)	1.04 (0.93–1.15)	
Yes	Reference	Reference	
**Received STI prevention counseling by a health professional**			0.85
No	1.08 (0.99–1.18)	1.01 (0.91–1.12)	
Yes	Reference	Reference	
**Had ≥ 2 CD4 or viral load tests in past year**			0.27
No	1.14 (1.05–1.24)	1.08 (0.96–1.21)	
Yes	Reference	Reference	
**Prescribed ART in past year**			0.19
No	1.16 (1.04–1.30)	1.12 (0.98–1.29)	
Yes	Reference	Reference	
**Used barrier birth control methods in past year**			0.18
No	1.10 (1.02–1.19)	1.07 (0.97–1.17)	
Yes	Reference	Reference	

Abbreviations: CI = Confidence interval; PR = unadjusted prevalence ratio; APR = Adjusted prevalence ratio; OB/GYN = Obstetrician/Gynecologist; STI = Sexually Transmitted Infection; ART = Antiretroviral medications.

Bold = significant at p < 0.05 level.

^1^Non-Hispanic white and other women were combined due to small sample sizes. Comparisons showed that the percentages were similar between the two groups.

**Poverty level = based on U.S. Department of Health and Human Services poverty guidelines as of 2013; http://aspe.hhs.gov/poverty/13poverty.shtml.

**Fig 1:**
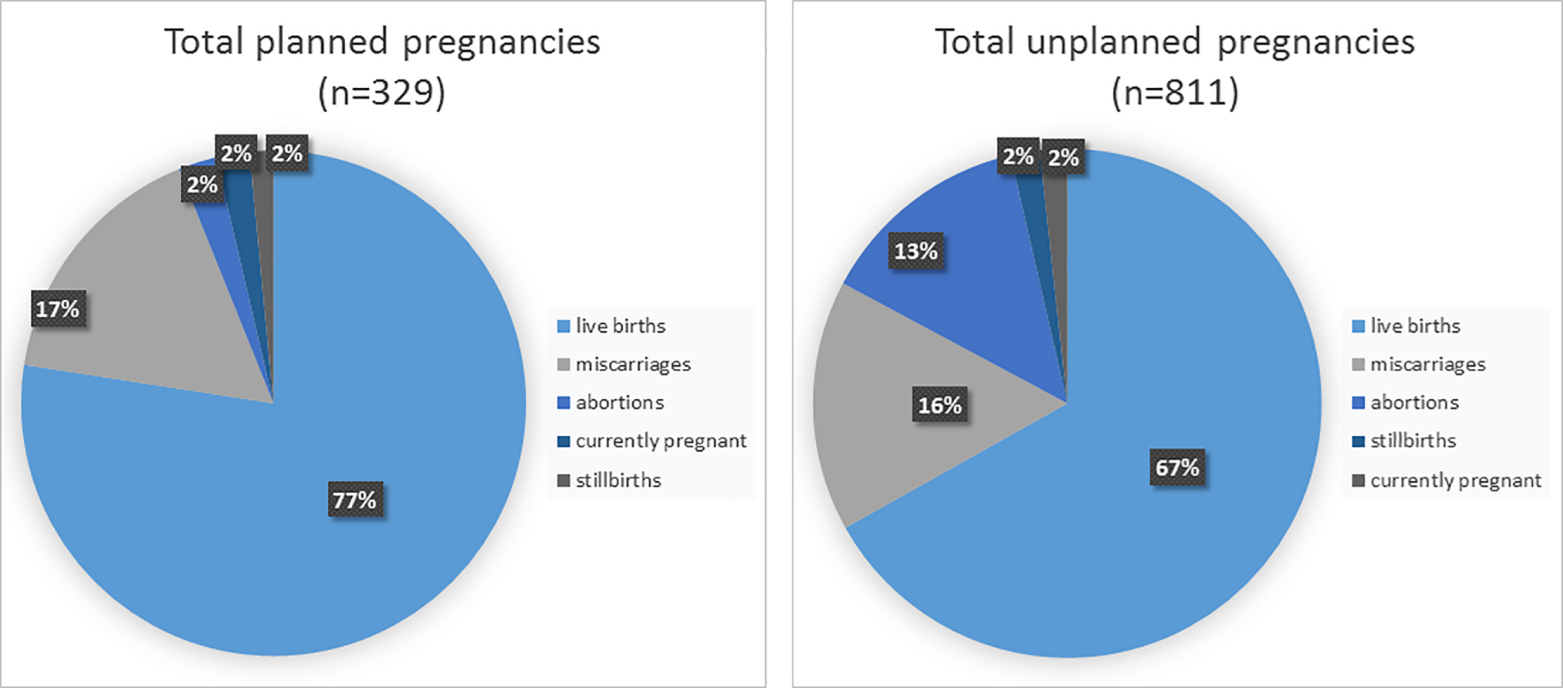
Percentage of each birth outcome among planned vs unplanned pregnancies, Medical Monitoring Project (n = 1,142).

Among the 1,142 total pregnancies, outcomes included 795 (69.6%) live births, 184 (16.1%) miscarriages, 119 (10.4%) abortions, and 20 (1.8%) stillbirths, and 21 (1.8%) were still pregnant at the time of the study (Fig 1). Of the live births, 717 (91.3%) infants were HIV-negative, 70 (7.8%) were HIV-positive, and 8 (1.0%) had indeterminate or unknown HIV status (data not shown). Of note, 42 (60%) HIV-positive babies were born to women reporting ≥ 1 unplanned pregnancies (data not shown). Fewer children were born HIV-positive among pregnancies occurring during and after 2004 (0.2% of live births) compared with those occurring before 2004 (13.2% of live births). Of children born HIV-positive, 100% of HIV-positive children were among unplanned pregnancies occurring during or after 2004, compared to 75% of those occurring before 2004; ART data are not available (whether the mothers were taking ART) for all babies born during the years reported by the women.

**Fig 2:**
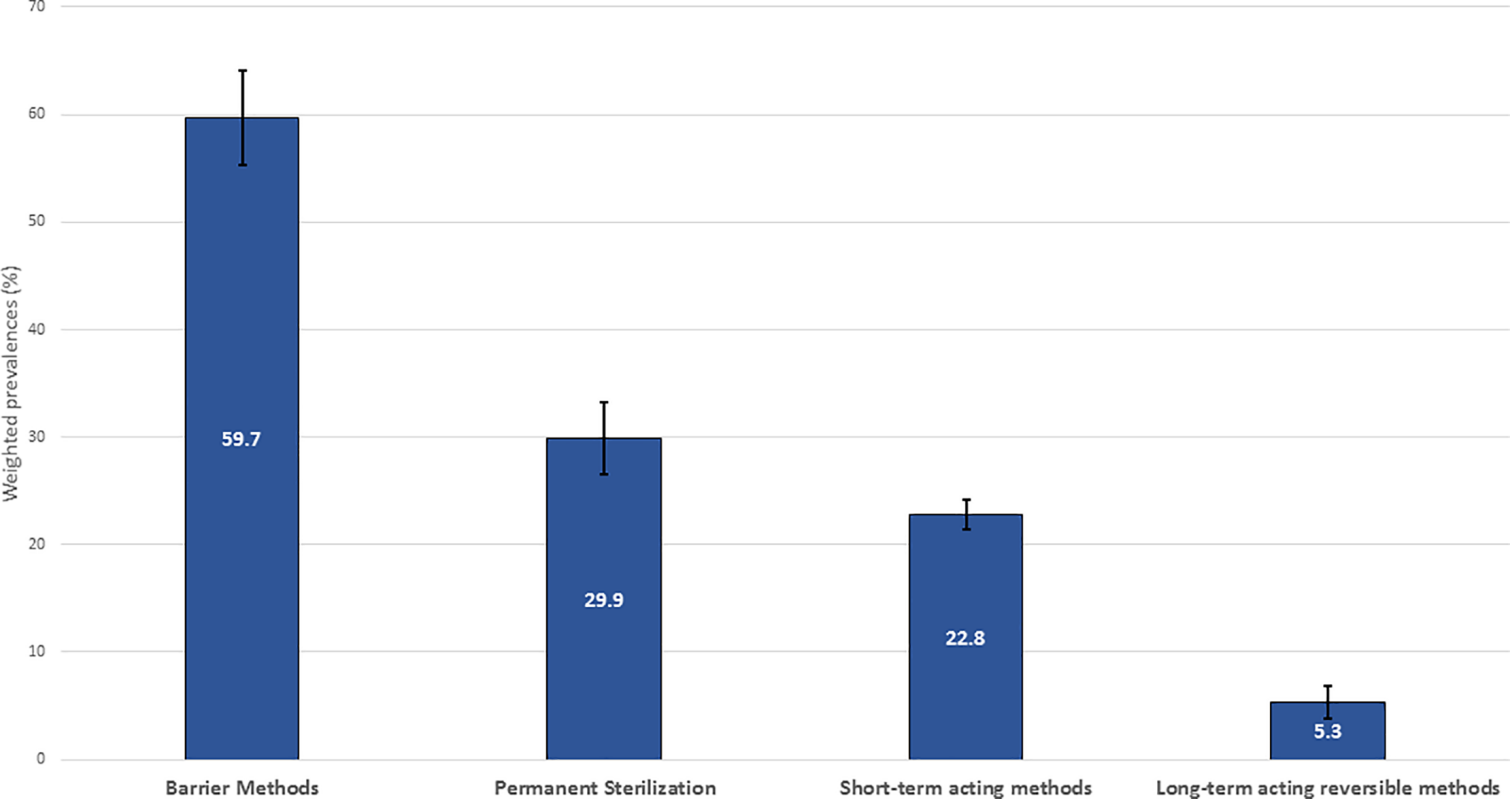
* Contraceptive use among HIV-positive women in HIV care aged 18–44 years, Medical Monitoring Project, 2013–2014 (n = 957). *Footnote: Methods may have been selected more than once. Graph excludes women who were not able to become pregnant due to hysterectomy and those who were abstinent.

There were 957 women who were interviewed while aged 18–44 years; 90.5% of them reported past-year contraceptive use (Fig 2). Fig 2 shows the distribution of contraceptives reported by the women, with barrier methods and permanent sterilization being more prevalent than short- acting methods and LARC. LARC methods were least frequently reported.

In multivariable analyses (Table 3), women reporting use of LARC or DMPA were significantly more likely to be aged 18–29 years (aPR = 3.08; CI 1.61–5.89) or 30–39 years (aPR = 2.86; CI 1.76–4.63) compared with women who were aged 40–44 years.

**Table 3:** Associations between selected characteristics and using highly effective, non-daily/weekly adherence contraception (LARC and DMPA) among HIV-positive women in care—Medical Monitoring Project, 2013–2014 (N = 654).

Characteristics	Unadjusted/Bivariate analyses	Adjusted/Multivariate analyses	
	PR (95% CI)	APR (95% CI)	p-value
**Race/Ethnicity**	** **	** **	
Non-Hispanic black	1.39 (0.70–2.78)		** **
Non-Hispanic white/Other^1^	1.29 (0.62–2.67)		** **
Hispanic	Reference		
**Age group at time of interview (years)**			**< .01**
18–29	3.15 (1.79–5.56)	3.08 (1.61–5.89)	
30–39	2.57 (1.70–3.88)	2.86 (1.76–4.63)	
40–44	Reference	Reference	
Poverty level**	** **	** **	0.23
Above poverty level	Reference	Reference	
At or below poverty level	1.29 (0.96–1.74)	1.29 (0.85–1.97)	
**Received care from OB/GYN in past year **			0.16
No	1.19 (0.77–1.84)	1.44 (0.83–2.51)	
Yes	Reference	Reference	
**Health insurance coverage **			0.12
Any private insurance	1.79 (0.86–3.75)	2.02 (0.90–4.56)	
Public insurance only	2.15 (1.10–4.21)	2.04 (1.02–4.08)	
Ryan White coverage only or uninsured			
**Any history of unplanned pregnancy since HIV diagnosis**			
1	Reference		
≥ 2	1.29 (0.63–2.64)		
**Had ≥ 2 CD4 or viral load tests in past year**			
No	Reference		
Yes	1.14 (0.69–1.90)		
**Prescribed ART in past year**			
No	1.33 (0.80–2.20)		
Yes	Reference		
**Used barrier birth control methods in past year**			0.07
No	Reference	Reference	
Yes	1.53 (0.96–2.43)	1.51 (0.96–2.37)	

Abbreviations: CI = Confidence interval; PR = unadjusted prevalence ratio; APR = Adjusted prevalence ratio; OB/GYN = Obstetrician/Gynecologist; STI = Sexually Transmitted Infection; ART = Antiretroviral medications.

Bold = significant at p < 0.05 level.

^1^Non-Hispanic white and other women were combined due to small sample sizes. Comparisons showed that the percentages were similar between the two groups.

**Poverty level = based on U.S. Department of Health and Human Services poverty guidelines as of 2013; http://aspe.hhs.gov/poverty/13poverty.shtml.

In sensitivity analyses, 59 of 671 or 8.4% of women in our sample had any pregnancies in the past year. Women with pregnancies in the past year did not differ significantly from women with any previous pregnancies in unplanned pregnancies (80.2% vs. 78.1%), currently taking ART (97.4% vs. 94.3%), and having sustained viral suppression (56.8% vs. 65.1%), respectively (data not shown), though cell sizes were small for women with pregnancies within the past year; these data should be interpreted with caution. For contraceptive methods used in the past year, 92.1% of women with any unplanned pregnancies during the past year and 89.9% of women reporting unplanned pregnancies at any time since their HIV diagnosis, reported using at least one form of contraception.

## Discussion

Thirty percent of reproductive-aged HIV-positive women in our study became pregnant at least once after their HIV diagnosis. Among women with at least one pregnancy, unplanned pregnancies were prevalent, reported by nearly 80% of women with any pregnancies. This suggests episodes of vaginal sex without contraception, or with suboptimal contraceptive barriers, a known risk factor for HIV acquisition or transmission (when there is detectable viral load) and unplanned pregnancies. For women with only planned pregnancies, recent care from an OB/GYN was more likely than for women who reported any unplanned pregnancies since their HIV diagnosis. We also found that >90% of women in our sample reported recent contraceptive use, whether or not they had had any pregnancies. However, barrier use, including condoms (for dual protection from pregnancies and ongoing sexual HIV/STI transmission), and LARC use (for effective, long-term contraception) were sub-optimal. These data highlight that there may be opportunities for improved reproductive health management during clinical care visits, including discussions of contraceptive options and pregnancy planning, for women living with HIV infection.

Clinically, women with no reported pregnancies were more likely to have recent sustained viral suppression compared with women with at least one pregnancy. Although the time windows for these variables are different, this data correlation is interesting based on reports showing viral suppression to be suboptimal among pregnant and post-partum women compared with non-pregnant women [25]. Previous studies suggest there may be a disengagement from HIV care during and possibly after pregnancy that may create challenges for HIV-positive women reaching viral suppression in the short and long-term [26].

Regarding live birth outcomes, of children born HIV-positive, more were from unplanned versus planned pregnancies. This finding was not related to date of pregnancy, although fewer children were born HIV-positive during and after 2004. While unplanned pregnancies are prevalent among reproductive-aged women regardless of HIV status, for HIV-positive mothers, perinatal HIV transmission is largely preventable using effective, evidence-based prevention strategies and with earlier pregnancy diagnosis and engagement in prenatal care. Data show that black/African American and Hispanic/Latino women and perinatally infected infants are disproportionately affected, representing 63.0% and 18.3% of mother-infant pairs, respectively [19]. Missed opportunities for early health care access, entry to care and other social determinants of health, disproportionately affect some women of color and play a major role in these alarming racial/ethnic disparities; culturally tailored strategies and resources are needed to reduce the burden of perinatal HIV transmission in communities of color [27].

For all women, having planned pregnancies allows for early engagement in obstetrical care and subsequent antiretroviral treatments, which would significantly decrease the chance of mother-to-child transmission [28]. This underscores the importance of assisting reproductive-age women in HIV care to engage in routine sexual health and family planning discussions with their providers so that their needs are met regarding access to effective contraception or access to early antenatal services, as patients’ needs may fluctuate over time [29]. As four-fifths (80.8%) of this representative sample of HIV-positive women in care have either public insurance, are covered by Ryan White or uninsured, helping women maintain access to health care, especially through publicly available and affordable health care, is a vital public health strategy for women.

Regarding contraceptives, we found that most women used some form of birth control methods in the past year. Barrier methods (condoms) were the most frequently reported form of contraception for women in our study, which is consistent with other reports [13]. The reported barrier use rate of 59.7% among women in our sample is lower than the 73.7% reported by HIV-positive women in WIHS [13]. Although these samples of different U.S. women are different, our data suggest room for improvement in condom use by HIV-positive women and as a dual protection strategy. Also consistent with a previous report of HIV-positive women [13], LARC use was reported by about 5% of women in our study. A recent study of privately insured HIV-positive women reported a 6.8% LARC usage rate [30]. Because LARC methods are highly effective, safe, and decrease daily, monthly or even quarterly patient compliance requirements, strengthening awareness and uptake of LARC use among women can be an additional important tool to decrease the high numbers of unplanned pregnancies; our study shows significantly greater use among younger, reproductive age women in our sample, which suggests increasing acceptability among women under age 40 years [29]. These findings have implications for condom reinforcement messages and other family planning and risk reduction strategies that can be used in clinical and non-clinical settings across all age groups.

## Limitations

Our study had some limitations. First, our MMP sample included only women who were in HIV care in the U.S.; findings may not apply to HIV-positive women who are not in HIV care. Engaging women who are not in care and women in other countries will be important for future analyses to best inform prevention efforts. Second, our data are limited by pregnancy dates not fully overlapping with contraceptive use dates or other characteristics such as viral suppression in the past 12 months; we recognize that contraceptive and sexual practices, as well as clinical care patterns observed in the past 12 months, may not reflect those at the time of pregnancies from more than 12 months previously. A longitudinal assessment of HIV-positive women and their pregnancy and/or contraceptive patterns is warranted to acquire more time-sensitive data for stronger comparative analyses. Third, asking participants to recall details of previous pregnancies may have led to recall bias. Fourth, social desirability bias may have affected participants’ disclosure about condomless sex with male partners and/or pregnancy outcomes. Fifth, nonresponse bias was possible. However, our sampling frame was based on a probabilistic sampling frame and allowed us to examine characteristics of sampled patients (sex, age, race, length of time since HIV diagnosis) to conduct a comparative analysis of respondents and non-respondents. Research has shown that well-constructed samples with moderate rates have reduced risk of bias.

## Conclusion

Among reproductive-aged, HIV-positive women in care, unplanned pregnancies are prevalent and use of highly effective LARC is low. Public health messaging regarding pregnancy planning, condomless sex, and options for use of long-term contraceptive methods, continue to be vital for all clinical providers, especially HIV and OB/GYN providers, to consider as part of routine clinical care.

## Supporting information

S1 Table Contraceptives interviewer question used in the Medical Monitoring Project, 2013–2014. (DOCX)

S2 Table Supplementary table. Selected Characteristics of HIV-positive Women in Care Who were Diagnosed Prior to Age 45 Years, Comparing by the Number of Pregnancies Since HIV Diagnosis—Medical Monitoring Project- 2013–2014, (N = 2228).
(DOCX)

S1 Fig Flow chart of samples for pregnancy and contraceptive analyses, Medical Monitoring Project (MMP), 2013–2014. (DOCX)
