# Supplementary material for: Unplanned pregnancies and contraceptive use among HIV- positive women in care
Source: PLoS One. 2018 May 17;13(5):e0197216. doi: 10.1371/journal.pone.0197216 (PMC5957391; doi:10.1371/journal.pone.0197216)
Supplement: S1 Table — (DOCX) [file pone.0197216.s001.docx]

**S1 Table.** **Contraceptives Interviewer Question used in the Medical Monitoring Project, 2013-2014**

Interviewer Text: I am going to read a list of ways that people prevent pregnancy. As I read each one, tell me if you have used it in the past 12 months. Please answer "YES" or "NO.” Answer “YES” even if you have only used it once. Have you used this method to prevent pregnancy in the past 12 months? ”

Male condom, also called a “rubber”

Female condom

Diaphragm, cervical cap, or cervical sponge

Spermicidal foam or jelly

Depo-Provera®, which is an injection

Hormonal implants such as Implanon® or Nexplanon®

Birth control pills

Contraceptive patch, for example, Ortho Evra®)

Contraceptive ring, for example NuvaRing®)

Intrauterine device or IUD, which comes as a coil or loop, for example, Mirena® or Paraguard®

Emergency contraception or “morning after pill”

Withdrawal, also called “pulling out”

Abstinence, which is not having sex

Post-menopausal, meaning that you do not have periods anymore

Tubal sterilization, which is having your “tubes tied,” or a hysterectomy, which is having your uterus removed

Partner’s vasectomy
