# Supplementary material for: Unplanned pregnancies and contraceptive use among HIV- positive women in care
Source: PLoS One. 2018 May 17;13(5):e0197216. doi: 10.1371/journal.pone.0197216 (PMC5957391; doi:10.1371/journal.pone.0197216)
Supplement: S2 Table — Selected Characteristics of HIV-positive Women in Care Who were Diagnosed Prior to Age 45 Years, Comparing by the Number of Pregnancies Since HIV Diagnosis—Medical Monitoring Project- 2013–2014, (N = 2228). (DOCX) [file pone.0197216.s002.docx]

**S2 Table. Selected Characteristics of HIV-positive Women in Care Who were Diagnosed Prior to Age 45 Years, Comparing by the Number of Pregnancies Since HIV Diagnosis - Medical Monitoring Project- 2013 – 2014, (N=2228)**

|  | **Total** | | **Had No Pregnancies** | | **Had >=1 Pregnancies** | | **Chi-Square** |
| --- | --- | --- | --- | --- | --- | --- | --- |
|  | **n** | **%†(95% CI)** | **n** | **% (95% CI)** | **n** | **% (95% CI)** | **p-value^** |
| **Total** | 2228 | 100 | 1557 | 69.8 (67.7-71.8) | 671 | 30.2 (28.2-32.3) |  |
| **Age group at time of diagnosis (years)** | | |  |  |  |  | **<0.01** |
| <19 | 173 | 8.3 (6.9-9.7) | 64 | 4.6 (3.3-5.9) | 109 | 16.8 (13.7-19.8) |  |
| 20-24 | 340 | 15.2 (13.6-16.8) | 148 | 9.3 (7.6-10.9) | 192 | 29.0 (25.9-32.1) |  |
| 25-29 | 419 | 19.1 (17.4-20.9) | 228 | 15.0 (13.2-16.8) | 191 | 28.8 (25.6-31.9) |  |
| 30-34 | 499 | 22.2 (20.2-24.2) | 376 | 24.1 (21.8-26.3) | 123 | 17.8 (14.7-20.8) |  |
| 35-39 | 443 | 19.3 (17.5-21.1) | 399 | 25.1 (22.9-27.3) | 44 | 5.9 (3.7-8.1) |  |
| 40-44 | 354 | 15.9 (14.2-17.7) | 342 | 22.0 (19.7-24.3) | 12 | 1.8 (0.5-3.1) |  |
| **Age group at time of interview (years)** | | |  |  |  |  | **<0.01** |
| 18-29 | 172 | 8.3 (6.8-9.7) | 90 | 6.2 (4.8-7.7) | 82 | 12.9 ( 9.8-16.0) |  |
| 30-39 | 413 | 19.0 (16.9-21.2) | 223 | 15.1 (12.6-17.5) | 190 | 28.2 (24.6-31.8) |  |
| 40-44 | 366 | 15.6 (13.9-17.3) | 214 | 13.2 (11.2-15.3) | 152 | 21.2 (17.7-24.7) |  |
| >45 | 1277 | 57.1 (54.6-59.5) | 1030 | 65.4 (62.9-68.0) | 247 | 37.7 (33.7-41.7) |  |
| **Race/Ethnicity** |  |  |  |  |  |  | 0.54 |
| Non-Hispanic black | 1363 | 61.3 (53.1-69.5) | 964 | 62.0 (53.2-70.8) | 399 | 59.7 (51.3-68.1) |  |
| Non-Hispanic white | 337 | 15.6 (12.5-18.7) | 238 | 15.9 (12.2-19.6) | 99 | 14.8 (10.5-19.1) |  |
| Hispanic** | 457 | 19.6 (11.2-28.0) | 304 | 18.6 (10.1-27.1) | 153 | 22.1 (13.2-31.0) |  |
| Other | 71 | 3.5 (2.1-4.8) | 51 | 3.5 (2.2-4.8) | 20 | 3.4 (1.4-5.4) |  |
| **Highest Educational Attainment** | |  |  |  |  |  | 0.66 |
| <High school | 651 | 29.5 (26.5-32.5) | 463 | 30.1 (27.0-33.2) | 188 | 28.3 (23.8-32.8) |  |
| High school graduate | 727 | 32.5 (30.0-35.1) | 512 | 32.5 (29.5-35.6) | 215 | 32.6 (28.5-36.6) |  |
| >High school | 849 | 37.9 (34.6-41.3) | 582 | 37.4 (33.6-41.2) | 267 | 39.2 (35.1-43.2) |  |
| **Health Insurance Coverage** | |  |  |  |  |  | 0.36 |
| Any private insurance | 415 | 19.1 (16.1-22.1) | 296 | 19.7 (16.4-23.1) | 119 | 17.8 (14.1-21.5) |  |
| Public insurance only | 1534 | 67.9 (62.7-73.2) | 1064 | 66.8 (61.0-72.6) | 470 | 70.5 (64.6-76.5) |  |
| Ryan White coverage only or uninsured | 278 | 12.9 (9.3-16.5) | 196 | 13.5 (9.1-17.8) | 82 | 11.6 (8.1-15.2) |  |
| **At or below poverty level** | 1472 | 68.8 (64.7-72.9) | 1009 | 67.5 (63.0-71.9) | 463 | 71.8 (66.6-77.1) | 0.09 |
| **Received care from OB/GYN** | 623 | 28.1 (22.1-34.1) | 395 | 25.0 (18.8-31.2) | 228 | 35.3 (28.5-42.1) | **<0.01** |
| **Currently taking ART** | 2103 | 94.6 (93.3-95.8) | 1472 | 94.8 (93.4-96.2) | 631 | 93.9 (92.1-95.8) | 0.39 |
| **Had > 2 CD4 or viral load tests** ^‡^ | 1915 | 86.3 (84.2-88.3) | 1340 | 86.3 (83.7-88.9) | 575 | 86.2 (83.4-89.0) | 0.96 |
| **Had sustained viral suppression** ^‡^ | 1443 | 64.6 (62.0-67.1) | 1038 | 66.5 (63.4-69.6) | 405 | 60.1 (56.4-63.8) | **0.007** |
| **Sexually active with a male partner (vaginal, anal, or oral sex)** | 1227 | 54.8 (52.5-57.0) | 768 | 49.4 (46.6-52.2) | 459 | 67.2 (63.1-71.4) | **<0.01** |
| **Sex risk/partner HIV status** | |  |  |  |  |  | **0.005** |
| Had condomless sex with HIV-negative or unknown status male partner | 306 | 24.7 (21.1-28.4) | 165 | 21.7 (17.7-25.7) | 141 | 29.9 (25.2-34.7) |  |
| Had condomless sex with HIV-positive male partner | 149 | 12.3 (10.4-14.2) | 98 | 12.7 (10.1-15.3) | 51 | 11.6 (8.6-14.6) |  |
| Did not report any condomless sex or not sexually active | 746 | 63.0 (58.9-67.0) | 490 | 65.6 (61.0-70.2) | 256 | 58.5 (53.4-63.6) |  |
| **Used any birth control methods** | | |  |  |  |  | 0.43 |
| Only one method used | 878 | 40.8 (38.4-43.1) | 611 | 41.3 (38.6-44.0) | 267 | 39.5 (35.3-43.8) |  |
| More than one method used | 1118 | 48.1 (45.0-51.2) | 778 | 47.2 (43.8-50.6) | 340 | 50.2 (45.2-55.1) |  |
| No method used | 232 | 11.1 (9.0-13.3) | 168 | 11.5 (9.1-13.9) | 64 | 10.3 (7.7-12.9) |  |
| **Used barrier birth control methods** | 1049 | 45.8 (42.2-49.4) | 669 | 41.7 (37.6-45.7) | 380 | 55.4 (50.6-60.2) | **<0.01** |
| **Data cycle year** |  |  |  |  |  |  | 0.10 |
| 2013 | 1142 | 53.4 (51.2-55.6) | 814 | 54.6 (52.1-57.0) | 328 | 50.7 (46.5-54.8) |  |
| 2014 | 1086 | 46.6 (44.4-48.8) | 743 | 45.4 (43.0-47.9) | 343 | 49.3 (45.2-53.5) |  |

Abbreviations: n = unweighted sample size; CI = Confidence interval; ART = Antiretroviral medications; VL = Viral Load; STI = Sexually Transmitted Infection; OB/GYN= Obstetrician/Gynecologist

Time period: In the past 12 months, unless otherwise noted. All measures are self-reported unless otherwise noted.

Bold=significant at p < 0.05 level.

^†^ weighted column percentage

^^^ Chi-square p-value based on the Rao-Scott chi-square

^‡^ Undetectable or < 200 copies/ml based on medical record abstraction data in the 12 months prior to interview.

^**^Hispanics or Latinos can be of any race.
