# Supplementary material for: Unplanned pregnancies and contraceptive use among HIV- positive women in care
Source: PLoS One. 2018 May 17;13(5):e0197216. doi: 10.1371/journal.pone.0197216 (PMC5957391; doi:10.1371/journal.pone.0197216)
Supplement: S1 Fig — (DOCX) [file pone.0197216.s003.docx]

S1 Fig. Flow chart of samples for pregnancy and contraceptive analyses, Medical Monitoring Project (MMP), 2013 – 2014

2013-2014 MMP sample

N=10,184

Women with only planned pregnancies (N=147)

Women with no pregnancies since HIV diagnosis (N=1,557)

Women with ≥ 1 pregnancy since HIV diagnosis (N=671*)

Unplanned pregnancy analysis (among women who had a pregnancy)

Contraceptive use analysis

Initial analytic sample

Women aged 18-44 years at time of interview (N=957)

Women with ≥ 1 unplanned pregnancy (N=524) (N=2,228)

Women diagnosed before age 45 years (N=2,228)

Total number of women in sample (N=2,766)

Total number of men in sample (N=7418)

*Among the 671 women with at least one pregnancy since their HIV diagnosis, there were 1,142 total pregnancies.
